# Rapid eco‐phenotypic feedback and the temperature response of biomass dynamics

**DOI:** 10.1002/ece3.9685

**Published:** 2023-01-10

**Authors:** Jean P. Gibert, Daniel J. Wieczynski, Ze‐Yi Han, Andrea Yammine

**Affiliations:** ^1^ Department of Biology Duke University Durham North Carolina USA

**Keywords:** biomass, temperature, temperature effects, temperature size rule, warming

## Abstract

Biomass dynamics capture information on population dynamics and ecosystem‐level processes (e.g., changes in production over time). Understanding how rising temperatures associated with global climate change influence biomass dynamics is thus a pressing issue in ecology. The total biomass of a species depends on its density and its average mass. Consequently, disentangling how biomass dynamics responds to increasingly warm and variable temperatures ultimately depends on understanding how temperature influences both density and mass dynamics. Here, we address this issue by keeping track of experimental microbial populations growing to carrying capacity for 15 days at two different temperatures, and in the presence and absence of temperature variability. We develop a simple mathematical expression to partition the contribution of changes in density and mass to changes in biomass and assess how temperature responses in either one influence biomass shifts. Moreover, we use time‐series analysis (Convergent Cross Mapping) to address how temperature and temperature variability influence reciprocal effects of density on mass and vice versa. We show that temperature influences biomass through its effects on density and mass dynamics, which have opposite effects on biomass and can offset each other. We also show that temperature variability influences biomass, but that effect is independent of any effects on density or mass dynamics. Last, we show that reciprocal effects of density and mass shift significantly across temperature regimes, suggesting that rapid and environment‐dependent eco‐phenotypic dynamics underlie biomass responses. Overall, our results connect temperature effects on population and phenotypic dynamics to explain how biomass responds to temperature regimes, thus shedding light on processes at play in cosmopolitan and abundant microbes as the world experiences increasingly warm and variable temperatures.

## Graphical abstract

We show that temperature and temperature fluctuations influence biomass dynamics through distinct effects on body size and density.

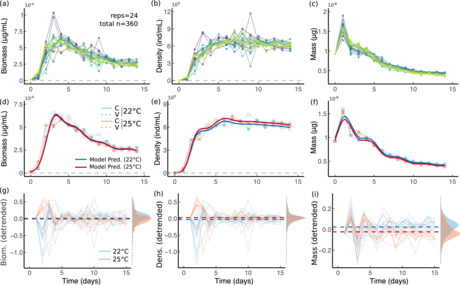

## 1 INTRODUCTION

Understanding the biotic and abiotic factors that influence ecosystem function is a central goal of Ecology (Begon et al., 2006; Giller & O'Donovan, 2002; Srivastava & Vellend, 2005). While censusing species presence/absence and abundances (or densities) provides a window into the overall structure of a community (e.g., composition, richness, evenness, and diversity), species abundances alone do not contain information on the ecosystem‐level functions performed by that community. However, tracking biomass (or biomass density) over time—i.e., the total mass of all individuals of a species or community (per unit area, if biomass density)—provides information on production within trophic levels, and comparing biomass across trophic levels can yield information on energy transfers within a food web (Barneche et al., 2021; D'Alelio et al., 2016; McKie & Malmqvist, 2009; Trebilco et al., 2013). Because of that, biomass is a central concept that both describes the state of an ecosystem and provides information on ecosystem‐level processes that influence overall functions like production or energy transfers (Hatton et al., 2015).

As the planet warms, the structural and dynamical responses of ecosystems are reflected in changes in biomass (Barbour & Gibert, 2021; Bartley et al., 2019; Gibert, 2019; Kortsch et al., 2015; Ullah et al., 2018). For example, the biomass of multiple taxa have been shown to decline with temperature across systems (Carr et al., 2018; Larjavaara et al., 2021; O'Connor et al., 2009). However, biomass declines are not universal (Lin et al., 2010) and the mechanisms through which warming influences species' biomasses are not well understood. Intuitively, because biomass is the sum of the mass of all individuals in a species, it is possible to decompose biomass into two main components: species' average masses and species' abundances (densities). Indeed, biomass is often estimated in the field as the product of the average mass of the individuals of a population and their abundance (or density). Consequently, any effects of temperature on biomass should, at their core, result from temperature effects on the abundance/density of a species or its average body size/mass.

Body size is an important functional trait that determines metabolic rates (Brown et al., 2004; Gillooly et al., 2001), demographic parameters (DeLong & Hanson, 2009; Savage et al., 2004; Wieczynski et al., 2021), species interactions (DeLong, 2014; DeLong et al., 2014, 2015), and even community and ecosystem‐level structure and processes (Allen et al., 2005; Gibert & DeLong, 2014; Schramski et al., 2015; Wieczynski et al., 2021). Increasing temperature generally reduces individual body sizes, an effect called the “temperature‐size rule” (TSR), which is pervasive across systems and taxa (Atkinson, 1994, 1995; Atkinson et al., 2003; Forster et al., 2012). For these reasons, body size and the temperature–size rule have clear consequences for changes in biomass across all levels of ecological organization in a warming world (Brose et al., 2012).

How temperature influences the other component of biomass—density—is less clear. The Metabolic Theory of Ecology predicts that warming should decrease species' carrying capacities (Savage et al., 2004)—the maximum density attainable in a given environment—but proof of that decline remained elusive until recently. Data‐tested theoretical work has now shown that while carrying capacity declines with temperature, this effect can only be observed by assuming both a direct effect of temperature on metabolic rates and the decline in body size with temperature, i.e., the TSR (Bernhardt et al., 2018). Moreover, while carrying capacities may indeed decline with temperature, it is unlikely that all species within a community will be at carrying capacity at any given moment—rather transient, non‐equilibrium dynamics are expected (Hastings et al., 2018). Thus, addressing whether and how non‐equilibrium densities are impacted by temperature is important for understanding how temperature influences biomass.

Last, body size can influence population growth, and hence densities, through relationships with demographic parameters like carrying capacity (K) and the intrinsic growth rate (r) (Damuth, 1981; DeLong et al., 2015; Savage et al., 2004). On the flip side, population dynamics could, in theory, also influence body size, through associated effects on resource levels, but these effects are less well understood. Recent work has shown that, as populations grow to carrying capacity, rapid changes in body size can have a stronger effect on changes in density than the other way around, suggesting an important—albeit asymmetric—feedback between population density and body size (Gibert et al., 2022). But how these reciprocal effects change with temperature, or how they may influence biomass responses to warming, is not known.

Here, we tackle these unknowns by addressing the following questions: (1) How is biomass affected by temperature and temperature variability as a species grows to carrying capacity? (2) To what extent are the effects of temperature on biomass dependent on how density and body size dynamics respond to temperature? (3) Does density or body size have a stronger effect on biomass responses to temperature? And, (4) do the reciprocal impacts of density and body size vary across temperature regimes? To address these questions, we recorded time series of population dynamics in a microbial species and tracked changes in total biomass, density, and body size in four different temperature regimes: constant 22°C, constant 25°C (i.e., a conservative +3°C warming scenario [IPCC, 2022]), and both temperatures with 3°C range fluctuations. We also derive a simple mathematical expression to partition the contribution of changes in density and body size to changes in biomass and assess how temperature responses in either one influence biomass shifts. Last, we use time‐series analyses to assess whether and how reciprocal effects of density and body size on biomass vary across temperature regimes.

## 2 METHODS

### 2.1 Study system

Microbial decomposers play a significant role in global biogeochemical process that fuel climate change (Zhou et al., 2011) as soil microbial respiration alone releases 98 Pg C.year^−1^ (Bond‐Lamberty & Thomson, 2010), thus representing one of the largest sources of atmospheric carbon emissions on the planet (Friedlingstein et al., 2019). The principal consumers of microbial decomposers worldwide are a group of single‐celled Eukaryotes collectively known as protists (Gao et al., 2019; Oliverio et al., 2020). For illustration, protists alone account for twice as much biomass as the entire animal kingdom globally (Bar‐On et al., 2018). Recently, predation has been shown to influence microbial decomposer biomass and determine their respiration rates (Geisen et al., 2021; Rocca et al., 2022). However, how this important group of organisms that regulates the global carbon cycle through predation may respond to rising temperatures is extremely poorly understood. In addition to their global importance, protists are easily grown in laboratory conditions (Altermatt et al., 2015) and have long been used to answer important ecological questions (Atkinson et al., 2003; DeLong & Hanson, 2009; Fox et al., 2011; Fronhofer et al., 2017; Geisen et al., 2018; Jassey et al., 2015; Wieczynski et al., 2021). Here, we study the biomass dynamics of the cosmopolitan and massively abundant (Finlay, 1998) bacterivore protist *Tetrahymena pyriformis*.

### 2.2 Microcosm growth assays

We grew populations of the protist *T. pyriformis* for 15 days (i.e., ~60 generations at roughly 4 generations per day) at various temperature treatments. The length of this experiment is a good compromise between ensuring the population spends time at carrying capacity (~10 days), and avoiding complete population collapse as resources are consumed. To do so, we set up 24 experimental microcosms in 250 ml autoclaved borosilicate jars containing 200 ml of Carolina protist pellet media (1 L of autoclaved DI water per pellet) previously inoculated with pond bacteria from Duke Forest (Gate 9/Wilbur pond, Lat = 36.02°, Long = −78.99°, Durham, NC) containing ~1000 bacterial ASVs (Rocca et al., 2022) and a wheat seed as a carbon source for the bacteria (Altermatt et al., 2015). All microcosms were started at 10 ind/ml protist densities and incubated in humidity‐controlled (65% humidity) growth chambers (Percival AL‐22L2, Percival Scientific, Perry, Iowa) on a 12 h night/day cycle. The entire replicated time series is therefore composed of 360 data points.

The 24 microcosms were subdivided into four experimental treatments: constant 22°C, constant 25°C, variable 22°C, or variable 25°C. The cultures have been growing in the laboratory for 3 years at constant 22°C prior to experimental work (>4000 generation), so 25°C represents a rapid +3°C temperature change relative to normal conditions (a conservative temperature estimate in the next 100 years [IPCC, 2022]). Temperature variability was programmed into our growth chambers to keep an average temperature, $\bar{T}$, of either 22°C or 25°C, and fluctuate between $\bar{T}+1.5{}^{\circ}C$ and $\bar{T}-1.5{}^{\circ}C$ every 12 h. The imposed thermal variability represents a significant departure from culturing conditions which see no temperature variability at all (temperature variability is also predicted to dramatically increase in new climates [IPCC, 2022]). All in all, a microcosm in the variable 22°C treatment thus spent half of the day at 19.5°C and half of the day at 23.5°C while one in the variable 25°C treatment spent half of the day at 23.5°C and half of the day at 26.5°C. At each temperature change, temperature ramped up/down for roughly 15 min. From now on we call these temperature treatments “constant” (C) and “variable” (V). Neither water nor nutrients were replaced throughout the course of this experiment.

### 2.3 Density, mass, and biomass estimates

**FIGURE 1:**
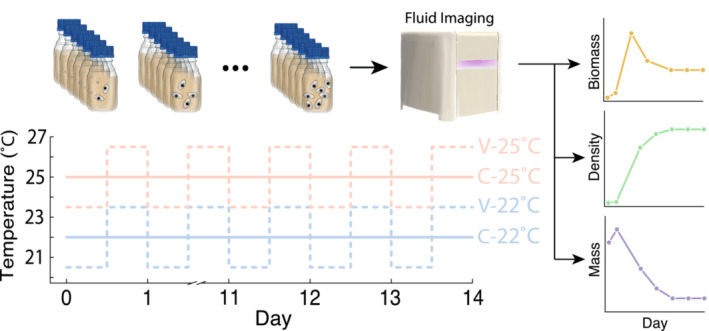
Microcosms were initialized at day 0 and kept in four possible temperature treatments (Constant 22°C, Variable 22°C, Constant 25°C, or Variable 25°C) for 15 days. Each day, a 1 ml sample of media was taken for fluid imaging (FlowCam) to estimate total biomass, density, and average mass as the species grew to carrying capacity.

Densities (ind/ml) and trait dynamics were tracked daily for 15 days through fluid imaging of 1 ml subsamples of each microcosm that had been shaken prior to sampling (Figure 1a, FlowCam, Fluid Imaging Technologies). The FlowCam captures images of particles ranging from 5–10 μm to 2 mm in length. The procedure produced ~450 k cell images, thus providing us with a unique window into how biomass, density, and body size, changed together over the course of this experiment. Density was quantified as cell counts per volume sampled. Cell mass was quantified as the product of cell volume (as the volume of a spheroid, in μm^3^) and water density (1 g/cm^3^, or 10–12 g/μm^3^). Sample biomass was measured as the sum of the masses of all individuals per sample (in grams, g). However, the FlowCam can only census a fraction of each water sample. This determines the efficiency of the machine (in our case ~0.33). Because of that, total biomass needs to be corrected by efficiency, as the observed number of individuals is a fraction of the total that actually occurs in our water samples. To do so, we linearly transform sample biomass according to the observed relationship between the number of cell images and the actual densities as detailed in Appendix S1. True biomass is therefore the observed biomass divided by the sampling efficiency.

### 2.4 Statistical analyses

To assess how temperature regimes influenced biomass, density, and mass dynamics, we used generalized additive mixed models (GAMMs) with biomass, density, or mass as the response variables, day as a smooth term, both temperature and the presence and absence of variation as discrete predictors, and jar replicate as a random intercept. Additionally, because time series are necessarily sampled in a repeated fashion within each replicate, temporal autocorrelation may exist. To account for this temporal autocorrelation, we included an autoregressive moving average (ARMA) correlation structure of order one in our GAMMs using the R package mgcv v.1.8 (Wood, 2011; Wood et al., 2016).

While GAMM yields a good understanding of how time and treatments influence dynamics, a finer understanding is possible by assessing what specific aspects of the dynamics may have been influenced by the treatments. First, we assessed whether the imposed treatments in any way influenced the peak observed biomass by running a multiple linear regression (“lm” function in base R [R Core Team, 2013]) with peak biomass (i.e., from days 3 to 5) as the response variable and both additive and interactive effects of temperature and the presence/absence of fluctuations as predictors. To quantify which differences between treatments were significant, we also ran a separate ANOVA with a post hoc Tukey test (“aov” and “TukeyHSD” functions in base R [R Core Team, 2013]) with peak biomass (i.e., from days 3 to 5) as the response variable and all four temperature treatments as separate predictors. We used the same statistical methods to assess whether demographic parameters controlling density—i.e., intrinsic growth rates, *r*, and carrying capacities, *K*—changed with treatment. Intrinsic growth rates *r* were calculated as the natural log of the ratio of the density at day 1 and the density at day 0 (Gibert et al., 2022; Wieczynski et al., 2021), and *K* was estimated as the densities measured over the last 2 days of the dynamics in each jar. Reported model results include coefficient estimates ± standard errors (SE) as well as associated test statistics and degrees of freedom.

### 2.5 Decomposing change in biomass into change in density and mass

To decompose the contribution of changes in density and mass to the observed changes in biomass, we assume that the biomass, *B*, could be written as a function of density, *N*, and average mass, *M*, as

(1) B=NM.

The rate of change in *B* over time, $\frac{\mathrm{dB}}{\mathrm{dt}}$, can be found by taking time derivatives on both sides of Equation (1), which yields:

(2) $$\frac{\mathrm{dB}}{\mathrm{dt}}=M\frac{\mathrm{dN}}{\mathrm{dt}}+N\frac{\mathrm{dM}}{\mathrm{dt}}.$$

We then noticed that Equation (1) could be used to solve for either *N* or *M*, as $N=\frac{B}{M}$ and $M=\frac{B}{N}$, and replaced both into Equation (2) to get:

(3) $$\frac{\mathrm{dB}}{\mathrm{dt}}=\frac{B}{N}\frac{\mathrm{dN}}{\mathrm{dt}}+\frac{B}{M}\frac{\mathrm{dM}}{\mathrm{dt}}.$$

Equation 3 could be further simplified by factoring *B*, dividing both sides of the expression by *B*, then using the relation $\frac{1}{x}\frac{\mathrm{dx}}{\mathrm{dt}}=\frac{d\;\mathrm{Ln}\left(x\right)}{\mathrm{dt}}$ to get:

(4) $$\frac{d\;\mathrm{Ln}\left(B\right)}{\mathrm{dt}}=\frac{d\;\mathrm{Ln}\left(N\right)}{\mathrm{dt}}+\frac{d\;\mathrm{Ln}\left(M\right)}{\mathrm{dt}}.$$

Equation 4 links the rate of change in $\mathrm{Ln}\left(B\right)$ to that of $\mathrm{Ln}\left(N\right)$ and $\mathrm{Ln}\left(M\right)$. This equation can thus be used to decompose the contributions of *N* (i.e., $\frac{d\;\mathrm{Ln}\left(N\right)}{\mathrm{dt}}$) and *M* (i.e., $\frac{d\;\mathrm{Ln}\left(M\right)}{\mathrm{dt}}$) to the rate of change in *B* over time and across temperature treatments. We used our experimental time series to calculate these contributions of *N* and *M* to changes in *B* for each individual jar on each day of the experiment.

### 2.6 Time‐series analysis

Previous studies have shown that convergent cross mapping (CCM) can be used to infer causation between variables with available time series across ecological systems and environmental conditions (Clark et al., 2015; Karakoç et al., 2020; Kondoh et al., 2020; Rogers et al., 2020; Sugihara et al., 2012). A recent study used CCM to show that rapid plastic change in body size has a larger effect on population dynamics, even though population dynamics also influence body size dynamics. This time‐series analysis was confirmed through a manipulative experiment (Gibert et al., 2022), therefore establishing the existence of an asymmetric effect of body size on density and of density on size. However, whether these reciprocal effects change in magnitude across environmental conditions is not known. We, therefore, used CCM to assess whether change in body size more strongly influenced changes in density, or vice versa, across temperature treatments.

Convergent cross mapping quantifies whether one time series (A) causally influences another (B) through the estimation of how much information of A is contained in B (Sugihara et al., 2012; Takens, 1981). Conceptually, if variable A causally influences variable B, but B does not influence A, then B should contain information about A, but not the other way around. CCM assesses how much information of the one variable is contained in the other by quantifying whether variable A can be predicted from the time series of B (and vice versa) for subsets of the time series of increasing length (the length of these resampled time series is called the library size). If A more strongly influences changes in B than the other way around, then B responds to A more strongly than A responds to B. If the effect of A on B is causal, then the ability to predict A from B increases with library size, while the error associated with the prediction decreases. If this “predictability” (or cross‐mapping skill, ρ) is constant across library sizes, there is correlation, but not causation (Sugihara et al., 2012). More details can be found in the now extensive literature on this algorithm (Barraquand et al., 2020; Brookshire & Weaver, 2015; Hannisdal et al., 2017; Kaminski et al., 2016; Liu et al., 2019; Luo et al., 2017; Mønster et al., 2017; Tsonis et al., 2018; Vannitsem & Ekelmans, 2018; Ye, Beamish, et al., 2015; Ye, Deyle, et al., 2015). We used a modified version of the CCM algorithm (R package multispatialCCM v1.0 [Clark et al., 2015]) to analyze the time series for each of the four temperature treatments because it allows for shorter replicated times series.

## 3 RESULTS

### 3.1 General dynamics

**FIGURE 2:**
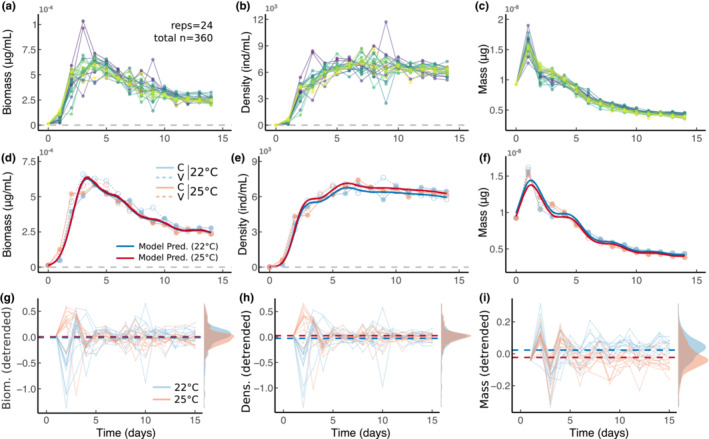
(a) Biomass over time for all 24 experimental jars. (b, c) as in (a) but for density and average mass, respectively. (d) Biomass change over time (dots represent average biomass across all six replicates within each experiment, blue represents 22°C treatments, red represents 25°C treatments, while solid lines represent constant temperature treatments, C, and dashed lines represent variable temperature treatments, (V). Solid bold lines represent GAMM model predictions. (e, f) as in (d) but for density and mass. (g) Detrended biomass dynamics across all temperature treatments (but only color coded for mean temperature as temperature variability had no effect in d–f) with color coding as in (d). Bold dashed lines represent mean biomass for both temperature treatments. The distribution that biomass values take over time are shown on the right. (h, i) as in (g) but for density and mass.

Biomass increased steeply in the early days of the dynamics, then declined over time (Figure 2) across temperatures. Density showed a typical logistic growth pattern of fast growth in the early days followed by a plateau at around 6000 ind/ml (Figure 2b). Average mass increased from Day 0 to Day 1, then decreased roughly monotonically over time (Figure 2c).

### 3.2 Effects of temperature and variability on biomass, density, and average mass

Biomass did not respond to either temperature (temp. effect = 0.02 ± 0.02 SE, *t* = 0.568, *df* = 8, *p* = .48, Figure 2d) or temperature variability (var. effect = −0.009 ± 0.02 SE, *t* = −0.271, *df* = 8, *p* = .73, Figure 2d). Temperature had a positive additive effect on density at 25°C relative to 22°C (temp. effect = 0.05 ± 0.02 SE, *t* = 2.37, *df* = 8, *p* = .018) while temperature variability had no effect (var. effect = 0.003 ± 0.02 SE, *t* = 0.142, *df* = 8, *p* = .89, Figure 2e). Temperature also had a negative effect on mass (temp. effect = −0.006 ± 0.003 SE, *t* = −5.812, *df* = 8, *p* = .002), but there was no effect of variability (var. effect = −0.03 ± 0.01 SE, *t* = −0.393, *df* = 8, *p* = .06, Figure 2f). These results suggest that the effects of temperature on density and mass likely cancel each other out, thus leading to an apparent lack of biomass temperature response.

Once the time series were detrended (by subtracting a GAMM model only containing time as a smooth term), additional effects of the treatments could be observed (Figure 2g–i). In particular, biomass and density showed similar strong effects of temperature (but not fluctuations) in the first few days of the dynamics (Figure 2g,h). Mass temperature responses, however, were most prevalent in the later dynamics, when the temperature size rule appears to set in (Figure 2i).

**FIGURE 3:**
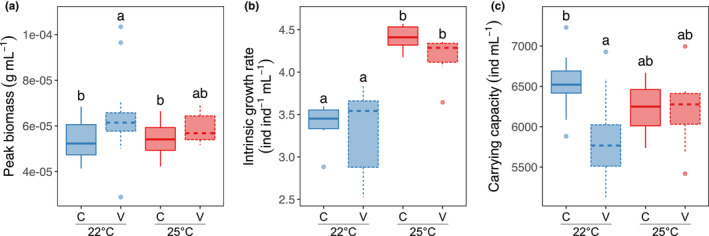
(a) Peak biomass (defined as the largest observed biomass in the days 3–5 bracket), with significant differences indicated as letters above the boxes. Variable temperatures lead to higher peak biomass, and that peak is higher at 22°C than at 25°C. (b) Intrinsic growth rate increases at 25°C. (c) Carrying capacity is higher at constant 22°C than in variable temperature but that difference disappears at 25°C. Blue = 22°C, red = 25°C, solid represent C treatments, and dashed represent V treatments.

Despite showing only transient effects of temperature and no effects of variability on overall biomass dynamics (Figure 2d–f), peak biomass in the variable environment was higher than in the non‐variable environment across temperatures, and this difference was only slightly smaller in the high‐temperature treatment, thus showing an effect of temperature variability but not temperature alone on peak biomass (temp. effect = 4 × 10^−7^ ± 3 × 10^−6^ SE, *t* = 0.118, *p* = .906, var. effect = 9.492 × 10^−6^ ± 3.410 × 10^−6^ SE, *t* = 2.784, *p* = .007, interaction = −5 × 10^−6^ ± 5 × 10^−6^ SE, *t* = −1.014, *p* = .314, *df* = 68; ANOVA, *F* = 3.43, *df* = 3, *p* = .02, Figure 3a). Temperature and temperature variability also influenced simple descriptors of what are otherwise complex density dynamics (Figure 3b,c). Indeed, temperature increased intrinsic growth rate despite fluctuations having no effect (temp. effect = 1.03 ± 0.02 SE, *t* = 5.14, *p* < 10^−4^, var. effect = −0.08 ± 0.02 SE, *t* = −0.376, *p* = .7, interaction = −0.17 ± 0.28 SE, *t* = −0.583, *p* = .6, *df* = 20; ANOVA, *F* = 15.43, *p* < 10^−4^, *df* = 3, Figure 3b; calculated using the first 2 days). Carrying capacity, on the other hand, decreased with variability but only at the low temperature and showed no significant differences in the high temperature (temp. effect = −304 ± 166 SE, *t* = −1.83, *p* = .074, var. effect = −696 ± 166 SE, *t* = −4.19, *p* < 10^−3^, interaction = 667.2 ± 235 SE, *t* = 2.84, *p* = .007, *df* = 44; ANOVA, *F* = 5.89, *p* = .002, *df* = 3, Figure 3c).

### 3.3 Decomposing the effects of density and mass on biomass across treatments

**FIGURE 4:**
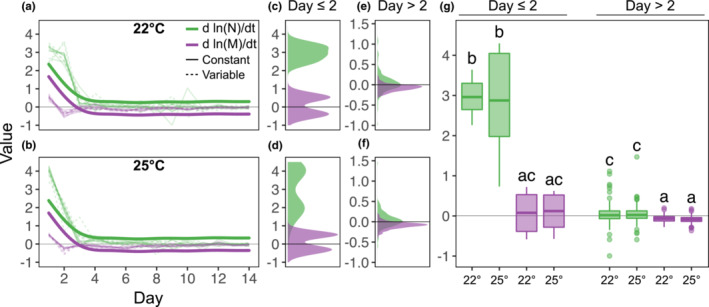
We depict the contributions of density (d ln(N)/dt, green) and mass (d ln(M)/dt, purple) to biomass dynamics at (a, c, e) 22°C and (b, d, f) 25°C, in constant (solid) and variable (dashed) environments. (a) and (b) show these contributions of density and mass change over time at 22°C and 25°C, respectively. Thin translucent lines represent replicate populations (jars) and thick lines are generalized additive mixed model (GAMM) fits to these data. (c, d) and (e, f) show distributions of density and mass contributions over time for days 0–2 (≤8 generations) and days >2 (>8 generations), respectively. (g) Shows differences in the contributions of density and mass across both temperatures for day ≤2 and day >2, evaluated using an ANOVA and Tukey's HSD post hoc test (*p* < 10^−5^).

Density and mass dynamics contributed distinctly to biomass dynamics, especially in the first 3 days (~12 generations, Figure 4). For day ≤2 (≤8 generations), rapid density increases strongly and positively influenced biomass, while mass only positively influenced biomass dynamics on day 1, then made mostly negative contributions (GAMM smooth term, edf = 7.87, Ref.*df* = 7.87, *F* = 72.32, *p* < 10^−16^, Figure 4a; ANOVA, *F* = 615, *p* < 10^−16^, *df* = 7, Figure 4g), likely due to the monotonous decline in mass from day 1 on (Figure 2c,f).

Despite temperature and temperature variability influencing both density and mass dynamics, their effects on the contributions of either one to biomass dynamics—i.e., $\frac{d\;\mathrm{Ln}\left(N\right)}{\mathrm{dt}}\;$ and $\frac{d\;\mathrm{Ln}\left(M\right)}{\mathrm{dt}}$—was surprisingly low, especially in the long term. Initially (day ≤2), density had a large, positive affect on biomass that remained high until day 2 at 22°C (Figure 4a) but declined sharply after day 1 at 25°C (Figure 4b, thin lines). Beyond day 2 (i.e., >8 generations), the contributions of either density or mass to biomass dynamics were small but different in sign (positive for density and negative for mass, Figure 4e–g). These results suggest that, while temperature treatment effects on biomass are most notable in the early dynamics, small, opposing effects of density and mass dynamics on biomass dynamics persist in the long term but are mostly unaffected by temperature and temperature fluctuations. Moreover, small temperature effects in the contributions of mass and density in the early dynamics are enough to produce larger effects later.

### 3.4 The temperature response of the coupling between density and mass

**FIGURE 5:**
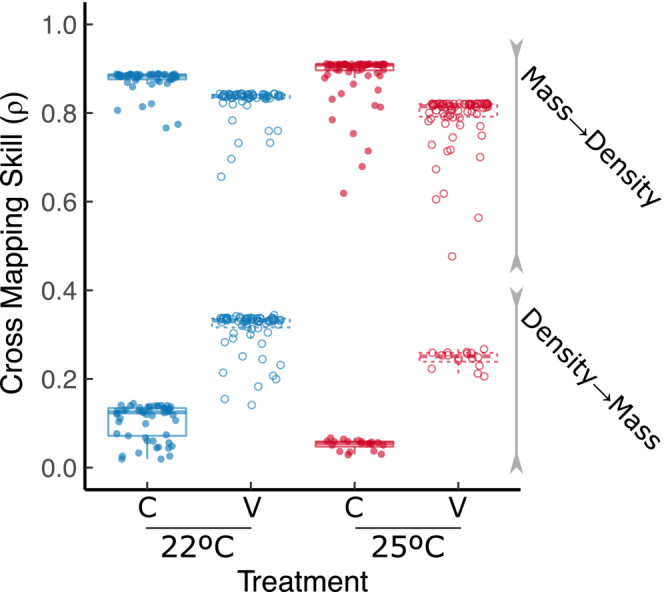
We show the cross‐mapping skill (ρ) for all possible library sizes across temperature treatments (here represented as individual dots). Package multispatialCCM performs CCM on 800 total bootstrap replicates of the time series for each library size and yields an average value for the cross‐mapping skill. Effects of mass on density are indicated as “Mass→Density” and effects of density on mass are indicated as “Density→Mass”. The effect of mass on density is larger than that of density on mass, but the reciprocal effects of mass and density respond to both average temperature and temperature fluctuations. Blue = 22°C, red = 25°C, solid represent C treatments, and dashed represent V treatments.

We observed that changes in mass more strongly influenced change in density than the other way around (consistent with a recent study [Gibert et al., 2022]) across all temperature treatments (Figure 5 and Figure S2 in Appendix S2). However, the strength of these reciprocal effects varied among treatments in specific ways. Temperature variability weakened the effect of mass on density across temperatures, and this effect was slightly stronger at 25°C compared to 22°C (Figure 5, temp. effect = 0.013 ± 0.001 SE, *t* = 1.581, *p* = .15, var. effect = −0.05 ± 0.009 SE, *t* = −5.327, *p* < 10^−6^, interaction = −0.05 ± 0.01 SE, *t* = −4.04, *p* < 10^−4^, *df* = 257). In contrast, the effect of density on mass weakened from 22°C to 25°C but got stronger with temperature fluctuations (Figure 5, temp. effect = −0.05 ± 0.01 SE, *t* = −5.21, *p* < 10^−6^, var. effect = 0.20 ± 0.007 SE, *t* = 29.77, *p* < 10^−16^, interaction = −0.02 ± 0.01 SE, *t* = −5.13, *p* = 0.29, *df* = 152). These results suggest that rapid feedback between density and mass (or “eco‐phenotypic feedback”) may themselves depend on environmental conditions—especially the effect of density on mass, which seems to respond more strongly to environmental variability than the effect of mass on density (Figure 5).

## 4 DISCUSSION

Understanding how changes in environmental conditions influence biomass dynamics is paramount in Ecology. Here, we argue that doing so requires understanding how temperature and temperature variability influence density and mass dynamics, then determining how those, in turn, influence biomass dynamics. Our results show that, while density and mass dynamics are independently susceptible to changes in temperature regimes (Figures 2 and 3), these effects may cancel each other out and not always translate to changes in biomass in response to temperature shifts (Figure 2). We also show that different aspects of density–mass–biomass dynamics respond differentially to variation in environmental conditions (Figure 3), even when environmental effects on overall dynamics are less obvious (Figure 2). We show that density and mass have mostly opposite effects on biomass and their contributions are nuanced and likely stronger in earlier dynamics (Figure 4). Last, we show that temperature and temperature variability can alter the strength of feedback between mass and density (Figure 5), suggesting that rapid eco‐phenotypic feedback may play an important but poorly understood role in biomass change in novel environments.

Temperature often reduces body size, a phenomenon widely known as the temperature–size rule (or TSR, e.g., [Atkinson, 1994, Atkinson et al., 2003]). This phenomenon is widespread, including among mammals (Ozgul et al., 2009), birds (Jirinec et al., 2021; Weeks et al., 2020), invertebrates (Ghosh et al., 2013), and unicellular organisms (Atkinson et al., 2003; DeLong, 2012; Tabi et al., 2020). The TSR has long been suggested to play an important role in the responses of populations (Ozgul et al., 2009), communities (Brose et al., 2012; Forster et al., 2012; Gibert & DeLong, 2017), and ecosystems (Brose et al., 2012) to warming, as changes in body size can directly impact reproductive and mortality rates and species interaction parameters through metabolic allometries (Brown et al., 2004; Gillooly et al., 2002; Savage et al., 2004). Our results show that the onset of the TSR occurs very early in population dynamics as species grow toward carrying capacity (Figure 1) and also suggest that, despite the numerous hypothesized effects of the TSR on ecological processes and dynamics, the TSR represents at most 5% of the observed variation in mass over time. Indeed, transient changes in mass are much larger in magnitude than the observed long‐term TSR (Figure 1), suggesting that rapid but transient shifts in body size, including in response to temperature, may be more important to understand ecological responses to a shifting environment than the TSR.

However, recent work has also shown that, without accounting for the TSR, predictions about how temperature influences long‐term species densities (i.e., at carrying capacity) may be inaccurate (Bernhardt et al., 2018). Our results further imply that, without accounting for the TSR, inferring changes in biomass from changes in density alone may lead to equivocal estimates, as the effects of temperature on density and mass can cancel each other out (Figure 2). These results are important because they imply that environmental perturbations may—sometimes rapidly—change populations not just numerically (e.g., changes in densities) but also phenotypically. Although the ecological consequences of these rapid, plastic, phenotypic responses are still very poorly understood, our results emphasize the need to improve this understanding.

Rapid, plastic changes in body size have recently been shown to more strongly influence changes in density (Gibert et al., 2022), thus establishing the existence of rapid but asymmetric feedback between body size and density driving ecological dynamics. This eco‐phenotypic feedback was also observed in our study. Additionally, our results show that the strength of this feedback varies across temperature regimes (Figure 4) and is differentially determined by both mean temperature and temperature variability. This result further emphasizes the need to understand rapid phenotypic change—evolutionary or not—as a fundamental ecological response mediating how species cope with novel environmental conditions.

While our results provide novel insights on how rapid eco‐phenotypic dynamics may mediate changes in biomass, density, and mass, in response to warming and temperature variability, we also acknowledge that a broader range of temperatures and temperature variability scenarios needs to be considered in future studies. Indeed, temperature effects are well known to have canonically unimodal effects on many demographic rates (Amarasekare & Coutinho, 2013; Amarasekare & Savage, 2012; DeLong et al., 2018; Luhring & DeLong, 2017; Wieczynski et al., 2021). Thus, eco‐phenotypic responses to a wider range of temperatures may be more complex than the results reported here imply. Moreover, the regimes of temperature fluctuations imposed here were less variable than the random fluctuations expected in an increasingly warmer world (Vasseur et al., 2014). Because of this, we caution against interpreting our results to say that average temperatures cause stronger species‐level responses than temperature variability. In fact, some of our results even suggest that variability does have important effects (Figures 3a and 5). Last, while CCM has long been used to infer effects of one time series on another (e.g. [Clark et al., 2015, Sugihara et al., 2012, Tsonis et al., 2018, Ye, Beamish, et al., 2015, Ye, Deyle, et al., 2015]), other unobserved variables like reductions in available nutrients, effects of regular sampling, or even physiological and metabolic changes as the ecological dynamics unfold may affect and even weaken the CCM inference. A silver lining is that our results are consistent with those obtained by Gibert et al. (2022), which were validated with additional body size and density manipulations and showed that CCM correctly inferred reciprocal effects between size and density based only on their time series, as was done here (Figure 5).

Overall, our results shed light on how rapid eco‐phenotypic dynamics in density and mass may influence how biomass responds to changes in temperature regimes. Our study emphasizes the need to consider rapid phenotypic change as an important—but poorly understood—mechanism through which organisms cope with changes in environmental conditions, with important implications for species' responses to a rapidly changing and increasingly warm world.

## AUTHOR CONTRIBUTIONS

**Jean Philippe Gibert:** Conceptualization (lead); data curation (supporting); formal analysis (lead); funding acquisition (lead); investigation (lead); methodology (equal); project administration (lead); writing – original draft (lead); writing – review and editing (lead). **Daniel Wieczynski:** Formal analysis (equal); investigation (supporting); methodology (supporting); writing – review and editing (equal). **Ze‐Yi Han:** Formal analysis (supporting); investigation (supporting); writing – review and editing (equal). **Andrea Yammine:** Conceptualization (supporting); data curation (lead); investigation (equal); project administration (supporting); writing – review and editing (supporting).

## FUNDING INFORMATION

This work was supported by a U.S. Department of Energy, Office of Science, Office of Biological and Environmental Research, Genomic Science Program Grant under Award Number DE‐SC0020362 to JPG.

## CONFLICT OF INTEREST

None.

### OPEN RESEARCH BADGES

This article has earned Open Data, Open Materials and Preregistered Research Design badges. Data, materials and the preregistered design and analysis plan are available at [https://github.com/JPGibert/Temperature_‐_Biomass_Dynamics].

## Supporting information


Appendix S1‐S2

## Data Availability

All data and code can be accessed for peer review and will be permanently stored at https://github.com/JPGibert/Temperature_‐_Biomass_Dynamics and on Zenodo (DOI: 10.5281/zenodo.7402022).

## References

[ece39685-bib-0001] Allen, A. P. , Gillooly, J. F. , & Brown, J. H. (2005). Linking the global carbon cycle to individual metabolism. Functional Ecology, 19, 202–213.

[ece39685-bib-0002] Altermatt, F. , Fronhofer, E. A. , Garnier, A. , Giometto, A. , Hammes, F. , Klecka, J. , Legrand, D. , Mächler, E. , Massie, T. M. , Pennekamp, F. , Plebani, M. , Pontarp, M. , Schtickzelle, N. , Thuillier, V. , & Petchey, O. L. (2015). Big answers from small worlds: A user's guide for protist microcosms as a model system in ecology and evolution. Methods in Ecology and Evolution, 6, 218–231.

[ece39685-bib-0003] Amarasekare, P. , & Coutinho, R. M. (2013). The intrinsic growth rate as a predictor of population viability under climate warming. Journal of Animal Ecology, 82, 1240–1253. 23926903 10.1111/1365-2656.12112

[ece39685-bib-0004] Amarasekare, P. , & Savage, V. M. (2012). A framework for elucidating the temperature dependence of fitness. The American Naturalist, 179, 178–191. 10.1086/663677 22218308

[ece39685-bib-0005] Atkinson, D. (1994). Temperature and organism size–a biological law for ectotherms? Advances in Ecological Research, 25, 1–58.

[ece39685-bib-0006] Atkinson, D. (1995). Effects of temperature on the size of aquatic ectotherms: Exceptions to the general rule. Journal of Thermal Biology, 20, 61–74.

[ece39685-bib-0007] Atkinson, D. , Ciotti, B. J. , & Montagnes, D. J. S. (2003). Protists decrease in size linearly with temperature: Ca. 2.5% degrees °C‐1. Proceedings of the Royal Society B: Biological Sciences, 270, 2605–2611. 10.1098/rspb.2003.2538 PMC1691543 14728784

[ece39685-bib-0008] Barbour, M. A. , & Gibert, J. P. (2021). Genetic and plastic rewiring of food webs under climate change. Journal of Animal Ecology, 90, 1814–1830. 34028791 10.1111/1365-2656.13541 PMC8453762

[ece39685-bib-0009] Barneche, D. R. , Hulatt, C. J. , Dossena, M. , Padfield, D. , Woodward, G. , Trimmer, M. , & Yvon‐Durocher, G. (2021). Warming impairs trophic transfer efficiency in a long‐term field experiment. Nature, 592, 76–79. 33647927 10.1038/s41586-021-03352-2

[ece39685-bib-0010] Bar‐On, Y. M. , Phillips, R. , & Milo, R. (2018). The biomass distribution on earth. Proceedings of the National Academy of Sciences of the United States of America, 115, 6506–6511. 29784790 10.1073/pnas.1711842115 PMC6016768

[ece39685-bib-0011] Barraquand, F. , Picoche, C. , Detto, M. , & Hartig, F. (2020). Inferring species interactions using granger causality and convergent cross mapping. Theoretical Ecology, 14, 87–105.

[ece39685-bib-0012] Bartley, T. J. , McCann, K. S. , Bieg, C. , Cazelles, K. , Granados, M. , Guzzo, M. M. , MacDougall, A. S. , Tunney, T. D. , & McMeans, B. C. (2019). Food web rewiring in a changing world. Nature Ecology & Evolution, 3, 345–354. 30742106 10.1038/s41559-018-0772-3

[ece39685-bib-0013] Begon, M. , Townsend, C. R. , & Harper, J. L. (2006). Ecology: From individuals to ecosystems (4th ed.). Blackwell Publishing.

[ece39685-bib-0014] Bernhardt, J. R. , Sunday, J. M. , & O'Connor, M. I. (2018). Metabolic theory and the temperature‐size rule explain the temperature dependence of population carrying capacity. American Naturalist, 192, 687–697. 10.1086/700114 30444656

[ece39685-bib-0015] Bond‐Lamberty, B. , & Thomson, A. (2010). Temperature‐associated increases in the global soil respiration record. Nature, 464, 579–582. 20336143 10.1038/nature08930

[ece39685-bib-0016] Brookshire, E. N. , & Weaver, T. (2015). Long‐term decline in grassland productivity driven by increasing dryness. Nature Communications, 6, 7148. 10.1038/ncomms8148 PMC4479003 25972300

[ece39685-bib-0017] Brose, U. , Dunne, J. A. , Montoya, J. M. , Petchey, O. L. , Schneider, F. D. , & Jacob, U. (2012). Climate change in size‐structured ecosystems. Philosophical transactions of the Royal Society of London. Series B, Biological Sciences, 367, 2903–2912. 10.1098/rstb.2012.0232 PMC3479741 23007078

[ece39685-bib-0018] Brown, J. H. , Gillooly, J. F. , Allen, A. P. , Savage, V. M. , & West, G. B. (2004). Toward a metabolic theory of ecology. Ecology, 85, 1771–1789.

[ece39685-bib-0019] Carr, L. A. , Gittman, R. K. , & Bruno, J. F. (2018). Temperature influences herbivory and algal biomass in the Galápagos Islands. Frontiers in Marine Science, 5, 1–10.29552559

[ece39685-bib-0020] Clark, A. T. , Ye, H. , Isbell, F. , Deyle, E. R. , Cowles, J. , Tilman, G. D. , & Sugihara, G. (2015). Spatial convergent cross mapping to detect causal relationships from short time series. Ecology, 96, 1174–1181. 26236832 10.1890/14-1479.1

[ece39685-bib-0021] D'Alelio, D. , Libralato, S. , Wyatt, T. , & Ribera D'Alcalà, M. (2016). Ecological‐network models link diversity, structure and function in the plankton food‐web. Scientific Reports, 6, 1–13. 26883643 10.1038/srep21806 PMC4756299

[ece39685-bib-0022] Damuth, J. (1981). Population density and body size in mammals. Nature, 290, 699–700.

[ece39685-bib-0023] DeLong, J. P. (2012). Experimental demonstration of a ‘rate – size’ trade‐off governing body size optimization. Evolutionary Ecology Research, 14, 343–352.

[ece39685-bib-0024] DeLong, J. P. (2014). The body‐size dependence of mutual interference. Biology Letters, 10, 20140261. 24919702 10.1098/rsbl.2014.0261 PMC4090548

[ece39685-bib-0025] DeLong, J. P. , Bachman, G. , Gibert, J. P. , Luhring, T. M. , Montooth, K. L. , Neyer, A. , & Reed, B. (2018). Habitat, latitude, and body mass influence the temperature dependence of metabolic rate. Biology Letters, 14, 20180442. 30158142 10.1098/rsbl.2018.0442 PMC6127111

[ece39685-bib-0026] DeLong, J. P. , Gilbert, B. , Shurin, J. B. , Savage, V. M. , Barton, B. T. , Clements, C. F. , Dell, A. I. , Greig, H. S. , Harley, C. D. G. , Kratina, P. , McCann, K. S. , Tunney, T. D. , Vasseur, D. A. , & O'Connor, M. I. (2015). The body size dependence of trophic cascades. The American Naturalist, 185, 354–366. 10.1086/679735 25674690

[ece39685-bib-0027] DeLong, J. P. , Hanley, T. C. , & Vasseur, D. A. (2014). Competition and the density dependence of metabolic rates. The Journal of Animal Ecology, 83, 51–58. 23565624 10.1111/1365-2656.12065

[ece39685-bib-0028] DeLong, J. P. , & Hanson, D. T. (2009). Metabolic rate links density to demography in Tetrahymena pyriformis. The ISME Journal, 3, 1396–1401. 19571891 10.1038/ismej.2009.81

[ece39685-bib-0029] Finlay, B. J. (1998). The global diversity of protozoa and other small species. International Journal of Parasitology, 28, 29–48. 9504333 10.1016/s0020-7519(97)00167-7

[ece39685-bib-0030] Forster, J. , Hirst, A. G. , & Atkinson, D. (2012). Warming‐induced reductions in body size are greater in aquatic than terrestrial species. Proceedings of the National Academy of Sciences of the United States of America, 109, 19310–19314. 23129645 10.1073/pnas.1210460109 PMC3511100

[ece39685-bib-0031] Fox, J. W. , Vasseur, D. A. , Hausch, S. , & Roberts, J. (2011). Phase locking, the Moran effect and distance decay of synchrony: Experimental tests in a model system. Ecology Letters, 14, 163–168. 21155962 10.1111/j.1461-0248.2010.01567.x

[ece39685-bib-0032] Friedlingstein, P. , Jones, M. W. , O'Sullivan, M. , Andrew, R. M. , Hauck, J. , Peters, G. P. , Peters, W. , Pongratz, J. , Sitch, S. , Le Quéré, C. , Bakker, D. C. E. , Canadell, J. G. , Ciais, P. , Jackson, R. B. , Anthoni, P. , Barbero, L. , Bastos, A. , Bastrikov, V. , Becker, M. , … Zaehle, S. (2019). Global carbon budget 2019. Earth System Science Data, 11, 1783–1838.

[ece39685-bib-0033] Fronhofer, E. A. , Nitsche, N. , & Altermatt, F. (2017). Information use shapes the dynamics of range expansions into environmental gradients. Global Ecology and Biogeography, 26, 400–411.

[ece39685-bib-0034] Gao, Z. , Karlsson, I. , Geisen, S. , Kowalchuk, G. , & Jousset, A. (2019). Protists: Puppet masters of the rhizosphere microbiome. Trends in Plant Science, 24, 165–176. 30446306 10.1016/j.tplants.2018.10.011

[ece39685-bib-0035] Geisen, S. , Hu, S. , Dela Cruz, T. E. E. , & Veen, G. F. C. (2021). Protists as catalyzers of microbial litter breakdown and carbon cycling at different temperature regimes. ISME Journal, 15, 618–621. 33005005 10.1038/s41396-020-00792-y PMC8027204

[ece39685-bib-0036] Geisen, S. , Mitchell, E. A. D. , Adl, S. , Bonkowski, M. , Dunthorn, M. , Ekelund, F. , Fernandez, L. D. , Jousset, A. , Krashevska, V. , Singer, D. , Spiegel, F. W. , Walochnik, J. , & Lara, E. (2018). Soil protists: A fertile frontier in soil biology research. FEMS Microbiology Reviews, 42, 293–323. 29447350 10.1093/femsre/fuy006

[ece39685-bib-0037] Ghosh, S. M. , Testa, N. D. , & Shingleton, A. W. (2013). Temperature‐size rule is mediated by thermal plasticity of critical size in Drosophila melanogaster. ZooKeys, 298, 20130174. 10.1098/rspb.2013.0174 PMC3652456 23595269

[ece39685-bib-0038] Gibert, J. P. (2019). Temperature directly and indirectly influences food web structure. Scientific Reports, 9, 5312. 30926855 10.1038/s41598-019-41783-0 PMC6441002

[ece39685-bib-0039] Gibert, J. P. , & DeLong, J. P. (2014). Temperature alters food web body‐size structure. Biology Letters, 10, 20140473. 25165457 10.1098/rsbl.2014.0473 PMC4155913

[ece39685-bib-0040] Gibert, J. P. , & DeLong, J. P. (2017). Phenotypic variation explains food web structural patterns. Proceedings of the National Academy of Sciences of the United States of America, 114, 11187–11192. 28973955 10.1073/pnas.1703864114 PMC5651739

[ece39685-bib-0041] Gibert, J. P. , Han, Z.‐Y. , Wieczynski, D. J. , Votzke, S. , & Yammine, A. (2022). Feedbacks between size and density determine rapid eco‐phenotypic dynamics. Functional Ecology, 36, 1668–1680.

[ece39685-bib-0042] Giller, P. S. , & O'Donovan, G. (2002). Biodiversity and ecosystem function: Do species matter? Biology and Environment: Proceedings of the Royal Irish Academy, 102B, 129–139.

[ece39685-bib-0043] Gillooly, J. F. , Brown, J. H. , West, G. B. , Savage, V. M. , & Charnov, E. L. (2001). Effects of size and temperature on metabolic rate. Science (New York, N.Y.), 293, 2248–2251. 11567137 10.1126/science.1061967

[ece39685-bib-0044] Gillooly, J. F. , Charnov, E. L. , West, G. B. , Savage, V. M. , & Brown, J. H. (2002). Effects of size and temperature on developmental time. Science, 417, 70–73. 10.1038/417070a 11986667

[ece39685-bib-0045] Hannisdal, B. , Haaga, K. A. , Reitan, T. , Diego, D. , & Liow, L. H. (2017). Common species link global ecosystems to climate change: Dynamical evidence in the planktonic fossil record. Proceedings of the Royal Society B, 284, 20170722. 28701561 10.1098/rspb.2017.0722 PMC5524498

[ece39685-bib-0046] Hastings, A. , Abbott, K. C. , Cuddington, K. , Francis, T. , Gellner, G. , Lai, Y.‐C. , Morozov, A. , Petrovskii, S. , Scranton, K. , & Zeeman, M. L. (2018). Transient phenomena in ecology. Science, 361, 1–9. 10.1126/science.aat6412 30190378

[ece39685-bib-0047] Hatton, I. A. , McCann, K. S. , Fryxell, J. M. , Davies, T. J. , Smerlak, M. , Sinclair, a. R. E. , & Loreau, M. (2015). The predator‐prey power law: Biomass scaling across terrestrial and aquatic biomes. Science, 349, aac6284. 26339034 10.1126/science.aac6284

[ece39685-bib-0048] IPCC . (2022). Climate change 2022: Impacts, adaptation, and vulnerability. Contribution of Working Group II to the Sixth Assessment Report of the Intergovernmental Panel on Climate Change Cambridge University Press.

[ece39685-bib-0049] Jassey, V. E. , Signarbieux, C. , Hattenschwiler, S. , Bragazza, L. , Buttler, A. , Delarue, F. , Fournier, B. , Gilbert, D. , Laggoun‐Defarge, F. , Lara, E. , Mills, R. T. , Mitchell, E. A. , Payne, R. J. , & Robroek, B. J. (2015). An unexpected role for mixotrophs in the response of peatland carbon cycling to climate warming. Scientific Reports, 5, 16931. 26603894 10.1038/srep16931 PMC4658499

[ece39685-bib-0050] Jirinec, V. , Burner, R. C. , Amaral, B. , Bierregard, R. O., Jr. , Fernández‐Arellano, G. , Hernández‐Palma, A. , Johnson, E. I. , Lovejoy, T. E. , Powell, L. L. , Rutt, C. , Wolfe, J. D. , & Stouffer, P. (2021). Morphological consequences of climate change for resident birds in intact Amazonian rainforest. Science Advances, 7, eabk1743. 34767440 10.1126/sciadv.abk1743 PMC8589309

[ece39685-bib-0051] Kaminski, M. , Brzezicka, A. , Kaminski, J. , & Blinowska, K. J. (2016). Measures of coupling between neural populations based on granger causality principle. Frontiers in Computer Sciences, 10, 114. 10.3389/fncom.2016.00114 PMC5080292 27833546

[ece39685-bib-0052] Karakoç, C. , Clark, A. T. , & Chatzinotas, A. (2020). Diversity and coexistence are influenced by time‐dependent species interactions in a predator‐prey system. Ecology Letters, 23, 983–993. 32243074 10.1111/ele.13500

[ece39685-bib-0053] Kondoh, M. , Kawatsu, K. , Osada, Y. , & Ushio, M. (2020). Theoretical ecology, concepts, and applications: A data‐driven approach to complex ecological systems. In K. S. McCann & G. Gellner (Eds.), Theoretical ecology, concepts and applications (pp. 117–133). Oxford University Press.

[ece39685-bib-0054] Kortsch, S. , Primicerio, R. , Fossheim, M. , Dolgov, A. V. , & Aschan, M. (2015). Climate change alters the structure of arctic marine food webs due to poleward shifts of boreal generalists. Proceedings. Biological Sciences/The Royal Society, 282, 20151546. 10.1098/rspb.2015.1546 PMC4571709 26336179

[ece39685-bib-0055] Larjavaara, M. , Lu, X. , Chen, X. , & Vastaranta, M. (2021). Impact of rising temperatures on the biomass of humid old‐growth forests of the world. Carbon Balance Management, 16, 31. 34642849 10.1186/s13021-021-00194-3 PMC8513374

[ece39685-bib-0056] Lin, D. , Xia, J. , & Wan, S. (2010). Climate warming and biomass accumulation of terrestrial plants: A meta‐analysis. New Phytologist, 188, 187–198. 20609113 10.1111/j.1469-8137.2010.03347.x

[ece39685-bib-0057] Liu, H. , Lei, M. , Zhang, N. , & Du, G. (2019). The causal nexus between energy consumption, carbon emissions and economic growth: New evidence from China, India and G7 countries using convergent cross mapping. PLoS ONE, 14, e0217319. 31120991 10.1371/journal.pone.0217319 PMC6532921

[ece39685-bib-0058] Luhring, T. M. , & DeLong, J. P. (2017). Scaling from metabolism to population growth rate to understand how acclimation temperature alters thermal performance. Integrative and Comparative Biology, 57, 103–111. 28662571 10.1093/icb/icx041

[ece39685-bib-0059] Luo, L. , Cheng, F. , Qiu, T. , & Zhao, J. (2017). Refined convergent cross‐mapping for disturbance propagation analysis of chemical processes. Computers & Chemical Engineering, 106, 1–16.

[ece39685-bib-0060] McKie, B. G. , & Malmqvist, B. (2009). Assessing ecosystem functioning in streams affected by forest management: Increased leaf decomposition occurs without changes to the composition of benthic assemblages. Freshwater Biology, 54, 2086–2100.

[ece39685-bib-0061] Mønster, D. , Fusaroli, R. , Tylén, K. , Roepstorff, A. , & Sherson, J. F. (2017). Causal inference from noisy time‐series data — Testing the convergent cross‐mapping algorithm in the presence of noise and external influence. Future Generation Computer Systems, 73, 52–62.

[ece39685-bib-0062] O'Connor, M. I. , Piehler, M. F. , Leech, D. M. , Anton, A. , & Bruno, J. F. (2009). Warming and resource availability shift food web structure and metabolism. PLoS Biology, 7, 3–8. 10.1371/journal.pbio.1000178 PMC2723928 19707271

[ece39685-bib-0063] Oliverio, A. M. , Geisen, S. , Delgado‐Baquerizo, M. , Maestre, F. T. , Turner, B. L. , & Fierer, N. (2020). The global‐scale distributions of soil protists and their contributions to belowground systems. Science Advances, 6, 1–10. 10.1126/sciadv.aax8787 PMC6981079 32042898

[ece39685-bib-0064] Ozgul, A. , Tuljapurkar, S. , Benton, T. G. , Pemberton, J. M. , Clutton‐Brock, T. H. , & Coulson, T. (2009). The dynamics of phenotypic change and the shrinking sheep of St. Kilda. Science (New York, N.Y.), 325, 464–467. 19574350 10.1126/science.1173668 PMC5652310

[ece39685-bib-0065] R Core Team . (2013). R: A language and environment for statistical computing. R Foundation for Statistical Computing.

[ece39685-bib-0066] Rocca, J. D. , Yammine, A. , Simonin, M. , & Gibert, J. P. (2022). Protist predation influences the temperature response of bacterial communities. Frontiers in Microbiology, 13, 847964. 35464948 10.3389/fmicb.2022.847964 PMC9022080

[ece39685-bib-0067] Rogers, T. L. , Munch, S. B. , Stewart, S. D. , Palkovacs, E. P. , Giron‐Nava, A. , Matsuzaki, S. S. , & Symons, C. C. (2020). Trophic control changes with season and nutrient loading in lakes. Ecology Letters, 23, 1287–1297. 32476249 10.1111/ele.13532 PMC7384198

[ece39685-bib-0068] Savage, V. M. , Gillooly, J. F. , Brown, J. H. , & Charnov, E. L. (2004). Effects of body size and temperature on population growth. The American Naturalist, 163, 429–441. 10.1086/381872 15026978

[ece39685-bib-0069] Schramski, J. R. , Dell, A. I. , Grady, J. M. , Sibly, R. M. , & Brown, J. H. (2015). Metabolic theory predicts whole‐ecosystem properties. Proceedings of the National Academy of Sciences of the United States of America, 112, 2617–2622. 25624499 10.1073/pnas.1423502112 PMC4345554

[ece39685-bib-0070] Srivastava, D. S. , & Vellend, M. (2005). Biodiversity‐ecosystem function research: Is it relevant to conservation? Annual Review of Ecology, Evolution, and Systematics, 36, 267–294.

[ece39685-bib-0071] Sugihara, G. , May, R. , Ye, H. , Hsieh, C. H. , Deyle, E. , Fogarty, M. , & Munch, S. (2012). Detecting causality in complex ecosystems. Science, 338, 496–500. 22997134 10.1126/science.1227079

[ece39685-bib-0072] Tabi, A. , Garnier, A. , Pennekamp, F. , & White, C. (2020). Testing multiple drivers of the temperature‐size rule with nonlinear temperature increase. Functional Ecology, 34, 2503–2512.

[ece39685-bib-0073] Takens, F. (1981). Detecting strange attractors in turbulence. Dynamical systems and turbulence. Springer.

[ece39685-bib-0074] Trebilco, R. , Baum, J. K. , Salomon, A. K. , & Dulvy, N. K. (2013). Ecosystem ecology: Size‐based constraints on the pyramids of life. Trends in Ecology & Evolution, 28, 423–431. 23623003 10.1016/j.tree.2013.03.008

[ece39685-bib-0075] Tsonis, A. A. , Deyle, E. R. , Ye, H. , & Sugihara, G. (2018). Convergent cross mapping: Theory and an example. In A. A. Tsonis (Ed.), Advances in nonlinear geosciences (pp. 587–600). Springer International Publishing AG. 10.1007/978-3-319-58895-7_27

[ece39685-bib-0076] Ullah, H. , Nagelkerken, I. , Goldenberg, S. U. , & Fordham, D. A. (2018). Climate change could drive marine food web collapse through altered trophic flows and cyanobacterial proliferation. PLoS Biology, 16, 1–21. 10.1371/journal.pbio.2003446 PMC5760012 29315309

[ece39685-bib-0077] Vannitsem, S. , & Ekelmans, P. (2018). Causal dependences between the coupled ocean–atmosphere dynamics over the tropical Pacific, the North Pacific and the North Atlantic. Earth System Dynamics, 9, 1063–1083.

[ece39685-bib-0078] Vasseur, D. A. , DeLong, J. P. , Gilbert, B. , Greig, H. S. , Harley, C. D. G. , McCann, K. S. , Savage, V. , Tunney, T. D. , & O'Connor, M. I. (2014). Increased temperature variation poses a greater risk to species than climate warming. Proceedings of the Royal Society B: Biological Sciences, 281, 20132612. 10.1098/rspb.2013.2612 PMC3924069 24478296

[ece39685-bib-0079] Weeks, B. C. , Willard, D. E. , Zimova, M. , Ellis, A. A. , Witynski, M. L. , Hennen, M. , & Winger, B. M. (2020). Shared morphological consequences of global warming in north American migratory birds. Ecology Letters, 23, 316–325. 31800170 10.1111/ele.13434

[ece39685-bib-0080] Wieczynski, D. J. , Singla, P. , Doan, A. , Singleton, A. , Han, Z. Y. , Votzke, S. , Yammine, A. , & Gibert, J. P. (2021). Linking species traits and demography to explain complex temperature responses across levels of organization. Proceedings of the National Academy of Sciences of the United States of America, 118, e2104863118. 34642248 10.1073/pnas.2104863118 PMC8545467

[ece39685-bib-0081] Wood, J. L. , Yates, M. C. , & Fraser, D. J. (2016). Are heritability and selection related to population size in nature? Meta‐analysis and conservation implications. Evolutionary Applications, 9, 640–657. 27247616 10.1111/eva.12375 PMC4869407

[ece39685-bib-0082] Wood, S. N. (2011). Fast stable restricted maximum likelihood and marginal likelihood estimation of semiparametric generalized linear models. Journal of the Royal Statistical Society B, 73, 3–36.

[ece39685-bib-0083] Ye, H. , Beamish, R. J. , Glaser, S. M. , Grant, S. C. , Hsieh, C. H. , Richards, L. J. , Schnute, J. T. , & Sugihara, G. (2015). Equation‐free mechanistic ecosystem forecasting using empirical dynamic modeling. Proceedings of the National Academy of Sciences of the United States of America, 112, E1569–E1576. 25733874 10.1073/pnas.1417063112 PMC4386326

[ece39685-bib-0084] Ye, H. , Deyle, E. R. , Gilarranz, L. J. , & Sugihara, G. (2015). Distinguishing time‐delayed causal interactions using convergent cross mapping. Scientific Reports, 5, 14750. 26435402 10.1038/srep14750 PMC4592974

[ece39685-bib-0085] Zhou, J. , Xue, K. , Xie, J. , Deng, Y. , Wu, L. , Cheng, X. , Fei, S. , Deng, S. , He, Z. , Van Nostrand, J. D. , & Luo, Y. (2011). Microbial mediation of carbon‐cycle feedbacks to climate warming. Nature Climate Change, 2, 106–110.

